# Internet gaming disorder and violent video gameplay among higher secondary school-going adolescents in Karachi, Pakistan—A cross-sectional study

**DOI:** 10.1371/journal.pmen.0000179

**Published:** 2025-01-03

**Authors:** Rabab Fatima Turabi, Shafquat Rozi, Sobiya Sawani, Momin Kazi, Nargis Asad

**Affiliations:** 1 Department of Community Health Sciences, Aga Khan University, Karachi, Pakistan; 2 Department of Pediatrics and Child Health, Aga Khan University, Karachi, Pakistan; 3 Department of Psychiatry, Aga Khan University, Karachi, Pakistan; Association for Socially Applicable Research (ASAR), INDIA

## Abstract

Problematic video game use is giving rise to psychiatric illnesses amongst adolescents including Internet Gaming Disorder (IGD). Violent content of video games can influence IGD development in vulnerable youth, yet its knowledge among Pakistani adolescents is limited, necessitating its assessment. This study estimated IGD prevalence among higher secondary school-going adolescents and assessed its association with violent video game content. A cross-sectional study was conducted on 286 school-going adolescents. Violent video game content was assessed using game ratings by the International Age Rating Coalition, while IGD, anxiety and depression were evaluated using Internet Gaming Disorder Scale–Short-Form (IGDS9-SF), Generalized Anxiety Disorder-7 (GAD-7) and Patient Health Questionnaire-9 (PHQ-9), respectively. Multiple Cox proportional algorithm was used to report adjusted prevalence odds ratios (POR) and 95% CI considering p-value of <0.05 as significant. Participants were predominantly males and from middle socioeconomic backgrounds. IGD prevalence was 17.50% (95% CI: 13.30–22.40). IGD and violent video game content had no association. However, IGD was associated with 3–4 hours of gaming on weekdays: POR = 5.295 (95% CI: 1.957–14.332), less than 7 years’ age of gaming onset: POR = 2.700 (95% CI: 1.035–7.046) and having a nuclear family: POR = 1.982 (95% CI: 1.021–3.844). Cronbach alpha for IGDS9-SF, GAD-7 and PHQ-9 was 0.742, 0.813 and 0.842 respectively. Findings suggest positive associations of IGD with prolonged gaming, early gaming onset and nuclear family. In conclusion, IGD is a growing concern for Pakistani adolescents. Findings suggest school-based awareness campaigns promoting healthier gaming practices and calls for government legislations for monitoring and control of age appropriate game usage.

## Introduction

Video gaming has become massively popular [1,2] as free-to-play games on app stores and gaming platforms, resonate with financially-dependent youth. Asia contributes to 45% of the global gaming community [3], including Pakistan which recorded approximately 32 million video game users in 2022 [4]. Rise in video game consumption prompted studies, linking gaming to Internet gaming disorder (IGD) among adolescents [5,6]. Defined as problematic gaming behavior that impairs gamers health, characterized by preoccupation, withdrawal, tolerance, inability to quit, disinterest in other hobbies, persistent gaming despite adverse effects, deceiving others regarding gaming duration, using games as an emotional outlet with or without increased risk taking behavior. Diagnosis requires persistent or recurring behavior over a year [7,8]. Global IGD prevalence ranges from 0.26% to 38% in adolescents with associated conditions including depression and anxiety [9]. Meta-analysis, reported pooled prevalence of 4.6% among adolescents [10]. Another study reported higher IGD prevalence linked to prolonged gaming, action genre and violent games [11]. In Pakistan, 1.5% of undergrads had IGD, reporting an association with game time and game genres of action and multiplayer-online-role-playing-games [12].

Popular games genres often feature violent content, described by the Entertainment Software Rating Board as "simulation of physical conflict, including injury, death, rape, and other acts of sexual violence involving humanoid characters with graphic depictions of blood, gore, and weaponry" [13]. Research indicates that exposure to violence in games can have adverse health effects [14–16]. Studies from India and Pakistan associate violent video games with higher frequency and duration of gameplay, as well as elevated IGD scores [12,17]. With growing popularity of gaming and majority of young aged demographic [18], Pakistani adolescents are at high risk for developing IGD. This study aimed to estimate the prevalence of IGD and assess its association with violent video games and other factors among higher secondary school-going adolescents in Karachi, Pakistan.

## Materials and methods

### Objectives

The study objectives were to estimate the prevalence of Internet gaming disorder and assess its association with violent video games among higher secondary school-going adolescents in Karachi, Pakistan.

### Study design

This study employed an analytical cross sectional design.

### Setting and site

This study was conducted in a higher secondary school based representative sample of Karachi, the largest metropolitan city in Pakistan, and site was one private and one government higher secondary school in Karachi, Pakistan. Each school had around 800 students, with male to female ratio of 1:1, enrolled in Intermediate or Advanced-Level classes.

### Study duration

Data collection was done in 2 months, in April and August 2023.

### Study population

Sample population was all higher secondary school going adolescents, attending the selected schools in Karachi, who fulfilled the eligibility criteria and provided informed written consent or assent and parental consent.

### Study eligibility criteria

Inclusion criteria were; adolescents i.e. aged between 10 to 19 years, enrolled in Intermediate or Advanced Level of education, attending the selected private or government higher secondary school, in Karachi, Pakistan, who agreed to participate in the study and gave their informed written consent or assent along with their parent/ guardians informed written consent regarding participation of their child in the study. Exclusion criteria were; who did not play a video game in the past 3 months, who did not provide at least one name of the video game or provided an unverifiable name, and who were not available during data collection.

### Sampling technique

We used a non-probability purposive sampling technique to select two higher secondary schools—one private and one government—based on their high enrollment rates and low absenteeism percentages, with the aim of maximizing data collection efficiency. Both schools included male and female students from diverse socioeconomic and cultural backgrounds, enhancing the generalizability of the study findings.

Data collection was conducted with the supervision and permission of the school administration after taking written informed consent from participants aged 18 years or older. Parental consent forms were sent to the parents/guardians of students below 18 years of age who were willing to participate via liaison with the school administration. However, due to non-response from parents/guardians, these students were not included in the study. Data collection was carried out at one point in time. There was no follow-up of the participants, and the recruitment period ended once they had filled out the questionnaire.

### Study variables

Data were collected using a paper based, self-administered questionnaire available in both English and Urdu languages, utilizing validated tools.

Outcome was IGD, treated as a binary variable. Assessed using the Internet Gaming Disorder Scale–Short-Form (IGDS9-SF) [19], that has 9 items rated on a 5 point Likert scale. Total score ranged from 9 to 45 and a score of ≥ 25 was termed as having IGD.

Primary exposure was violent video game content. The International Age Rating Coalition (IARC) rates all commercially available video games from a set of five rating levels [20]. Each level corresponds to an age for which its content is suitable and has content descriptors for violent and sexual content of the game as follows;

*“3+: Suitable for all ages. Some violence in comical or fantasy context is acceptable. Bad language is not permitted.”*

*“7+: May contain some scenes or sounds frightening for children. Mild violence (implied or non-realistic) is permitted.”*

*“12+: Violence involving fantasy characters with or without non-graphic violence involving human-looking characters or animals is permitted. Non-graphic nudity, mild language and simulated gambling are also permitted, but sexual expletives are not.”*

*“16+: Realistic violence, sexual activity, strong language, use of tobacco and drugs, and the depiction of criminal activities are permitted.”*

*“18+*: *Graphic violence*, *including depictions lacking motive and/or directed towards defenseless characters*, *and sexual violence are permitted*. *May also include graphic sexual content*, *discriminatory acts and /or the glamorization of illegal drug use*.*”*

These ratings were merged to create the exposure variable categorized as; non-violent = video game with ‘IARC rating 3+’, mild to moderately violent = video game with ‘IARC rating 7+ and 12+’ and severely violent = video game with ‘IARC rating 16+ and 18+’. Participants were asked to name the three most frequently played video games in the last three months. Game names were used to identify their IARC rating. If any one of the games played had a rating higher than ‘3+’, participant was considered exposed. Exposure was then sub-classified into ‘mild to moderately violent’ or ‘severely violent’ content based on the game with highest IARC rating.

Other covariates assessed were; participants age, gender, type of school, year of education, study course, Father/guardians and mother’s education and occupation, family type, school performance and attendance, age of onset of playing video games, frequently used gaming mode, motive for playing video games, frequently used gaming device and duration of gaming defined as average number of gaming hours per week for weekends and weekdays. Anxiety was assessed using the Generalized Anxiety Disorder-7 (GAD-7) tool [21]. It has 7 items rated on a 4 point Likert scale. The total score ranges from 0 to 21 and a score of ≥ 5 was labeled as having anxiety. Depression was assessed using the Patient Health Questionnaire (PHQ-9) tool [21]. It has 9 items rated on a 4 point Likert scale with total score range from 0 to 27. Score of ≥ 5 was labeled as having depression. Duration of playing video games, anxiety, depression and socioeconomic status were also assessed as potential confounders.

### Biases

To mitigate influence of social desirability or fear of repercussions on responses, self-administered questionnaires were used. They utilized neutral language, avoiding leading questions. Participants were assured of the confidentiality and anonymity of their data which reduced Hawthorne effect and raised participation rates minimizing volunteer bias. To reduce recall bias, questions regarding gaming asked about recent 3 months. Validated tools reduced detection bias. Inclusion of participants from both private and public schools ensured a socioeconomically diverse sample enhancing representativeness and raising generalizability of results, boosting external validity.

### Sample size

Keeping the ratio of adolescents playing nonviolent video games to adolescents playing violent video games as 1:2, the percentage of adolescents with IGD not exposed to violent video games as 16.4% [11], the expected prevalence odds ratio as 2.5 [22], and an inflation of 5% to accommodate for refusals and non-responses, a sample size of 286 was calculated to achieve 80% power and a 5% level of significance using Open Epi software version 3.01.

### Statistical analysis

Multiple cox proportional hazard algorithm was used to analyze data with a binary outcome of chronic nature for this cross-sectional study.

Contingency tables for all qualitative variables were made with IGD and categories showing data sparsity were merged. Information regarding household wealth, income, occupation and education was collected to determine participant’s socioeconomic status using factor analysis. Since participants were financially dependent on their parents/legal guardians as is the cultural practice in Pakistan, this information was collected regarding their parents/legal guardians. Simple cox regression was used to perform univariate analysis. Each covariate was regressed independently with IGD with cutoff for determining significance kept at ≤0.25. Mulitcollinearity was assessed using Crammers V test keeping a cutoff of ≥0.8 to determine its presence. Multivariable analysis using multiple cox regression was conducted using a stepwise model building approach. Variables significant at univariate level and not showing mulitcollinearity, including the primary exposure variable were included in the multivariable model. Keeping an inclusion cutoff of p-value of <0.05. A parsimonious model was built using cox proportional algorithm by adding those variables into the model that had the strongest association with IGD tested during univariate analysis. As the model was extended, the likelihood ratio tests were observed to determine if the model was significantly improving. All plausible interactions were assessed between violent video games and other independent variables. In the absence of any significant interaction effect, confounding was assessed, none of which was found. Adjusted prevalence odds ratios along with their 95% confidence intervals were reported to compare IGD with respect to violent video games and other factors and a p-value of <0.05 was treated as significant. All analysis was carried out on STATA software (version 15.0).

### Ethics statement

Approval received from Aga Khan University’s Ethics Review Committee (No.2023-8324-24477).

Written informed consent was obtained from participants aged 18 years and above. For those under 18 years, written informed assent and parental consent were sought; however, lack of response from their parents/guardians resulted in them not being included in the study. Consent forms were available in both English and Urdu. Every participant was given a copy of the signed consent forms. All participants were informed about the study’s objectives, methodology, any potential benefits or risks, maintenance of data confidentiality and privacy, as well as their right to refuse and withdraw at any time.

## Results

**Fig 1:**
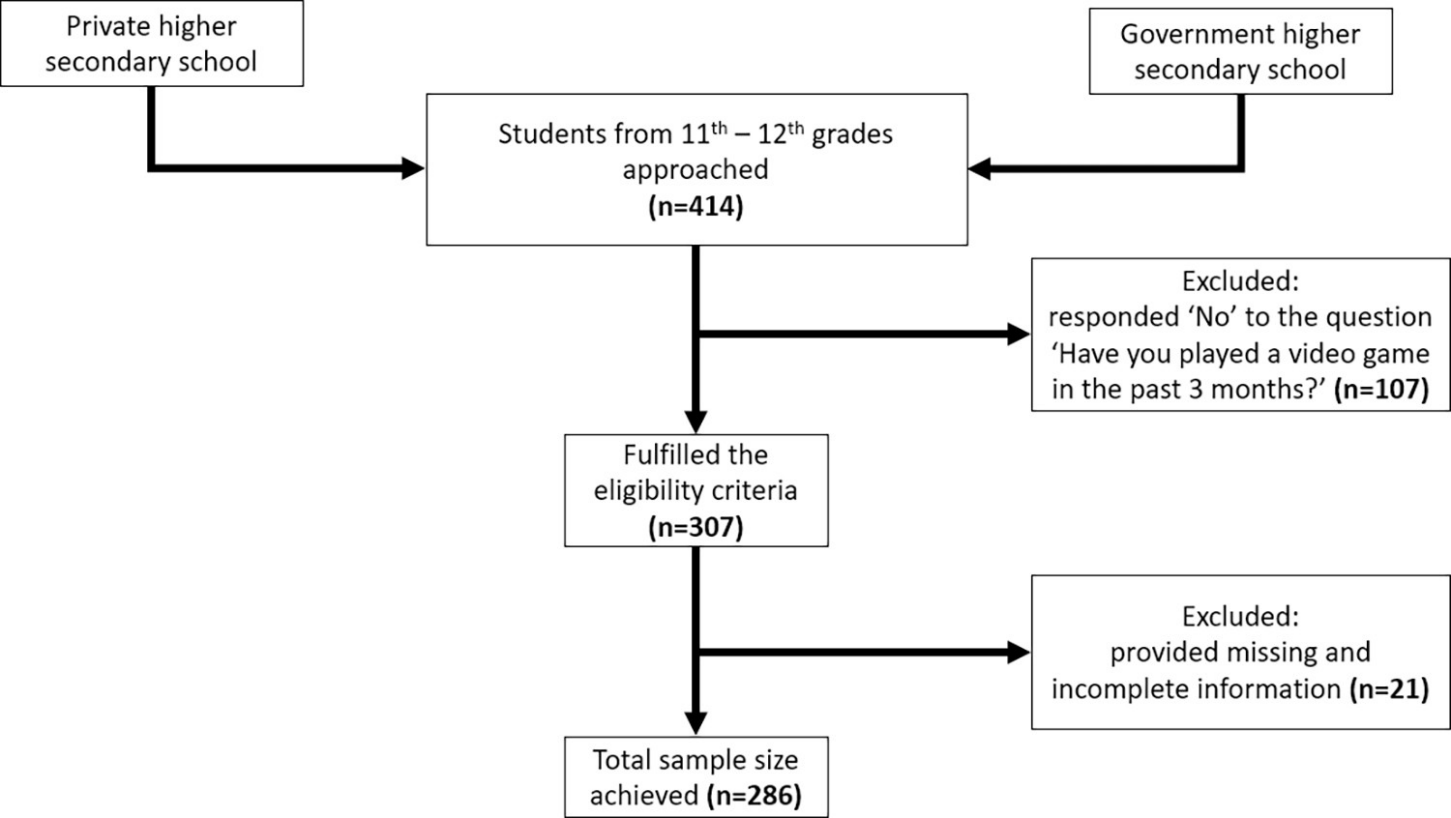
Participant recruitment flowchart.

A total of 414 students from 11th and 12th grades, attending the selected private and government higher secondary schools, were approached. After scrutinizing for eligibility criteria and addressing missing information, 286 students participated in the study (Fig 1).

Prevalence of Internet gaming disorder among higher secondary school going adolescents in urban areas of Karachi, Pakistan was 17.48% with a 95% confidence interval of 13.26–22.39%. Sociodemographic characteristics presented in Table 1 showed that more than half of the participants were males (73.78%) and 18 years of age (67.48%). As per the study’s methodology, equal number of participants were selected from private and government schools. Since the government school offered education specific to commerce course, all government school students’ course of study was ‘commerce’ (50%). While the private school was not subject specific. Most participants (60.49%), reported having a nuclear family.

**Table 1:** Sociodemographic characteristics and academic profile by Internet Gaming Disorder (IGD) of higher secondary school-going adolescents in Karachi, Pakistan (n = 286).

Characteristics	Total: n (%)	IGD present: n (%)
**Age (in years)**		
18 19	193 (67.48) 93 (32.52)	38 (19.69) 12 (12.90)
**Gender**		
Female Male	75 (26.22) 211 (73.78)	17 (22.67) 33 (15.64)
**Type of school**		
Private Government	143 (50.00) 143 (50.00)	32 (22.38) 18 (12.59)
**Year of education**		
11^th^ grade 12th grade	162 (56.64) 124 (43.36)	28 (17.28) 22 (17.74)
**Study course**		
Pre-medical Commerce Other	46 (16.08) 199 (69.58) 41 (14.34)	10 (21.74) 31 (15.58) 9 (21.95)
**School performance**		
Above average Average	116 (40.56) 170 (59.44)	17 (14.66) 33 (19.41)
**School attendance**		
Regular Absent more than or equal to once a week	212 (74.13) 74 (25.87)	31 (14.62) 19 (25.68)
**Father/ guardians education**		
University education Higher secondary school education Less than higher secondary school education	137 (47.90) 72 (25.17) 77 (26.92)	24 (17.52) 17 (23.61) 9 (11.69)
**Mothers education**		
University education Higher secondary school education Less than higher secondary school education	100 (34.97) 77 (26.92) 109 (38.11)	20 (20.00) 12 (15.58) 18 (16.51)
**Father’s/ guardians occupation**		
Not currently working Employed Self-employed	27 (9.44) 145 (50.70) 114 (39.86)	4 (14.81) 19 (13.10) 27 (23.68)
**Mothers occupation**		
Unemployed or housewife Other	244 (85.31) 42 (14.69)	44 (18.03) 6 (14.29)
**Family type**		
Joint Nuclear	113 (39.51) 173 (60.49)	12 (10.62) 38 (21.97)
**Socioeconomic status**		
Low Middle High	59 (20.63) 173 (60.49) 54 (18.88)	9 (15.25) 28 (16.18) 13 (24.07)

Regarding gaming behavior of participants presented in Table 2, more than half of them played video games with severely violent content (55.59%). The majority had started playing video games between the ages of 11 and 15 years (43.71%). Gaming duration on weekdays was similarly distributed for ‘1 hour or less’ and ‘between 1 to 2 hours’ (34.27%), while majority played ‘between 1 to 2 hours’ (32.87%) on weekends. Gaming duration increased from weekdays to weekends for the following categories; ‘between 2 to 3 hours’ (16.08% to 19.93%), ‘between 3 to 4 hours’ (5.94% to 6.99%) and for ‘4 hours or more’ (9.44% to 12.59%). Mobile devices including mobile phones and tablets were mostly used for gaming (63.29%), while 19.58% of the participants used more than one device to play video games. Online games (78.67%) were preferred and common motive for playing was ‘competition’ (28.32%). With regards to distribution of mental illnesses, more than half of the students experienced ‘anxiety’ (55.94%) and ‘depression’ (62.94%).

**Table 2:** Gaming related behavior and psychological characteristics by Internet Gaming Disorder (IGD) of higher secondary school-going adolescents in Karachi, Pakistan (n = 286).

Characteristics	Total: n (%)	IGD: n (%)
**Video game content**		
Non-violent Mild to moderately violent Severely violent	30 (10.49) 97 (33.92) 159 (55.59)	7 (23.33) 12 (12.37) 31 (19.50)
**Age of onset of playing video games (in years)**		
Above 15 11 to 15 7 to 10 Less than 7	64 (22.38) 125 (43.71) 65 (22.73) 32 (11.19)	8 (12.50) 19 (15.20) 12 (18.46) 11 (34.38)
**Duration of gaming on weekdays (in hours)**		
1 hour or less Between 1 to 2 Between 2 to 3 Between 3 to 4 4 hours or more	98 (34.27) 98 (34.27) 46 (16.08) 17 (5.94) 27 (9.44)	10 (10.20) 13 (13.27) 10 (21.74) 8 (47.06) 9 (33.33)
**Duration of gaming on weekends (in hours)**		
1 hour or less Between 1 to 2 Between 2 to 3 Between 3 to 4 4 hours or more	79 (27.62) 94 (32.87) 57 (19.93) 20 (6.99) 36 (12.59)	8 (10.13) 14 (14.89) 10 (17.54) 6 (30.00) 12 (33.33)
**Type of gaming device**		
Mobile devices Traditional gaming and computing platforms More than one device	181 (63.29) 49 (17.13) 56 (19.58)	32 (17.68) 8 (16.33) 10 (17.86)
**Gaming mode**		
Offline Online	61 (21.33) 225 (78.67)	9 (14.75) 41 (18.22)
**Motive for playing video games**		
Fantasy Skill development Competition Entertainment Respite More than one motive	73 (25.52) 57 (19.93) 81 (28.32) 28 (9.79) 25 (8.74) 22 (7.69)	12 (16.44) 11 (19.30) 10 (12.35) 6 (21.43) 6 (24.00) 5 (22.73)
**Anxiety**		
Absent Present	126 (44.06) 160 (55.94)	19 (15.08) 31 (19.38)
**Depression**		
Absent Present	106 (37.06) 180 (62.94)	12 (11.32) 38 (21.11)

IGD prevalence was examined according to participant characteristics as well, presented in Tables 1 and 2. Among females, 22.67% had IGD, while 15.64% of males reported IGD. From students enrolled in private schools, 22.38% had IGD, while from government schools 12.59% had IGD. Among those absent more than once a week, 25.68% had IGD, while those with regular attendance had an IGD prevalence of 14.62%. Participants having a nuclear family type and high socioeconomic status had IGD prevalence of 21.97% and 24.07%, respectively. Among those who played severely violent video games, 19.50% had IGD, while among those who played mild to moderately violent and non-violent video games, 12.37% and 23.33% had IGD, respectively. Those who started playing video games before the age of 7 years showed an IGD prevalence of 34.38%. Participants gaming for 3–4 hours on weekdays reported IGD prevalence of 47.06%. Among those who screened positive for anxiety, the prevalence of IGD was 19.38%, while for those screening positive for depression, IGD prevalence was 21.11%.

Upon univariate analyses unadjusted prevalence odds ratios and 95% CI were calculated. The following significant variables; age, gender, school attendance, father/guardian education, family type, age of onset of gaming, duration of gaming on weekdays, duration of gaming on weekends and depression, along with the primary exposure variable i.e. ‘video game content’, were included in multivariable analyses. No biologically plausible interaction or confounding was found. Adjusted and unadjusted prevalence odds ratios and their 95% confidence intervals and significance levels were reported in Table 3 to compare IGD with respect to violent video games and other covariates, considering a p-value of <0.05 as significant.

**Table 3:** Unadjusted and adjusted prevalence odds ratio with 95% CI of factors associated with IGD among higher secondary school-going adolescents in Karachi, Pakistan (n = 286).

Variable	Unadjusted POR (95% CI)	p-value	Adjusted POR (95% CI)	p-value
**Video Game content**				
Non–violent (reference)	-	-	-	-
Mild to moderately violent	0.530 (0.209–1.347)	0.182	0.491 (0.185–1.304)	0.154
Severely violent	0.836 (0.368–1.898)	0.668	0.537 (0.220–1.313)	0.173
**Duration of gaming on weekdays**				
1 hour or less (reference)	-	-	-	-
Between 1–2 hours	1.300 (0.570–2.965)	0.533	1.463 (0.624–3.428)	0.381
Between 2–3 hours	2.130 (0.887–5.118)	0.091	2.174 (0.876–5.396)	0.094
Between 3–4 hours	4.612 (1.820–11.685)	0.001	5.295 (1.957–14.332)	0.001*
4 hours or more	3.267 (1.327–8.039)	0.010	2.581 (0.972–6.849)	0.057
**Age of onset**				
More than 15 years (reference)	-	-	-	-
Between 11 to 15 years	1.216 (0.532–2.778)	0.643	1.101 (0.972–6.849)	0.824
Between 7 to 10 years	1.477 (0.604–3.613)	0.393	1.122 (0.450–2.797)	0.805
Less than 7 years	2.75 (1.106–6.837)	0.029	2.700 (1.035–7.046)	0.042*
**Family type**				
Joint (reference)	-	-	-	-
Nuclear	2.068 (1.081–3.958)	0.028	1.982 (1.021–3.844)	0.043*

*p-value of <0.05 considered as significant.

Violent video game content was insignificant at multivariable analyses, failing to reject the null hypothesis. While variables of gaming duration on weekdays of 3–4 hours, gaming age of onset of less than 7 years and having a nuclear family were significant at p-value of <0.05. It was reported that odds of IGD among higher secondary school-going adolescents who played video games for 3–4 hours on a weekday were 5.30 times compared to those who played for 1 hour or less on a weekday. While, odds of IGD among higher secondary school-going adolescents with age of gaming onset of <7 years were 2.7 times compared to those who began gaming after 15 years. Also, odds of IGD among higher secondary school-going adolescents having a nuclear family were 1.98 times compared to those belonging to a joint family.

## Discussion

Prevalence of IGD was 17.48% (95% CI: 13.26 and 22.39%), much higher than that reported by another Pakistani study on undergraduate students, estimating prevalence of 1.5% [12]. Our findings were more similar to a study in China reporting prevalence of 17% among adolescent gamers. Its larger sample size provided greater generalizability and accuracy of results, even though they utilized a different tool for IGD measurement [23]. A range of IGD prevalence among adolescent gamers has been reported from 0.26 to 38% [9,10] attributing it to differences in measurement tools and population demographics. Our study was thus in line with other Asian studies and also within range provided by global studies.

Our study had a higher proportion of male participants as compared to females, which can be explained by existing literature that reports males having a greater inclination towards gaming [24]; hence they were more likely to fulfill the inclusion criteria. Although recent statistics suggest a growing interest in video gaming among females in Western countries [25,26], attributed to higher female preference for social networking sites [27,28], as many video games are integrated into these platforms. Consequently, women in our region may also be exposed to a higher number of video games, potentially increasing the number of female gamers in the future.

Our study reported greater IGD prevalence among female video game players compared to males, contrary to existing literature [29]. Multivariable analysis however did not show gender as a significant predictor of IGD when adjusted for other sociodemographic factors and gaming behaviors, suggesting that the observed results could be driven by unknown confounders. Also, the smaller proportion of female participants in our study resulting in their underrepresentation could have hindered the ability to detect any significant gender effects on IGD. A study on Chinese university students provided evidence on females being more vulnerable to IGD but the differences in study population could not generalize the findings on Pakistani school-going adolescents [30]. There is a gap in existing literature addressing IGD among female adolescents in Pakistan. Future research efforts exploring female gaming preferences and behaviors in this region are warranted to confirm our study findings and identify potential factors that may contribute to this discrepancy.

Studies have associated game genres, such as First-Person Shooter (FPS), Role Playing Game (RPG), strategy, Massively Multiplayer Online Role Playing Game (MMORPG) and action, with an increased likelihood of problematic gaming [31–35]. This classification, however, is based on the type of player-game interaction and game setting. Video games vary greatly even within their particular genres making it difficult to isolate the exact cause of IGD. Despite the availability of internationally recognized standardized set of violent game ratings few studies have compared games based on their violence ratings when assessing the relationship between violent content and IGD. Research conducted in a closely similar population stated higher likelihood of IGD when playing violent video games [11], while another reported no evidence of a relationship between game genre and problematic gaming among adolescent gamers [36]. Given the limited existing literature and mixed findings, drawing a definitive conclusion is challenging. Further exploration of addictive game features, such as in-game reward systems, alongside socio-cultural factors such as family dynamics, home and school environments, and individual factors influencing attitudes and behaviors toward gaming may provide a better understanding of their potential role in moderating the relationship between violent video games and IGD.

Adolescents playing video games for longer durations were more likely to have IGD. This was consistent with findings of global studies, such as that reporting a significant association between gaming duration of more than 3 hours and IGD in Saudi Arabia [37], an Italian study on online gamers [38] and another on secondary school students in Brazil [39]. In addition, the “IGD Working Group of the National Academy of Sciences Colloquium on Digital Media and Developing Minds” stated that longer gaming durations could lead to IGD development among children and adolescents [40].

A positive association between early age of gaming onset and IGD was found in our study. A longitudinal study concluded that preschool exposures to video games increased IGD risk during adolescence [41]. Another study in Japan observed that children who began playing video games before 5 years of age were more likely to develop IGD later in life [42]. Early childhood exposure to videogames is therefore a likely predictor of disordered gaming later in life.

According to our study, participants having a nuclear family were twice as likely to have IGD. Pakistani culture endorses joint family structure, increasing chances of familial interactions. More interaction however does not always mean positive interaction. A sour relation with parents can increase likelihood of mental illnesses among children [43]. So the nature of relationship with one’s family is a predictor of mental illnesses. Since our study did not assess the relationship between adolescents and their parents, we cannot comment on whether the observed association was because of an unhealthy parent-child relationship.

In our study, over half of the participants screened positive for anxiety (55.94%), with an even higher percentage screening positive for depression (62.94%). Among those with anxiety, a higher proportion were males (64.38%) compared to females. Similarly, in terms of depression, more males (67.78%) were affected. Females, however, reported greater prevalence for both anxiety (76%) and depression (77.33%) compared to males (anxiety = 48.82%, depression = 57.82%), highlighting that females are more likely to experience these mood disorders, as supported by literature [44,45]. Associations between IGD and anxiety and depression have been reported in the past [46,47], suggesting that gaming offers an escape from real-life problems and can serve as a coping mechanism. However, our study could not establish such an association, possibly due to lack of severity assessments for these disorders and not adjusting for individual, environmental and socio-cultural factors differing between male and female adolescents, which may have confounded the association.

### Strengths

Tools validated among a Pakistani adolescent population were used. Questionnaires were available in English and Urdu to facilitate comprehension and reduce information loss. Content validation of questionnaire was done by adolescent psychiatry and psychology specialists. Cronbach alpha of tools used was as follows; 0.7429 for IGDS9-SF, 0.8130 for GAD-7 and 0.8420 for PHQ-9, indicating moderate to good level of internal consistency. Using the multiple cox-proportional hazard algorithm while keeping time constant to estimate the POR provided more precise effect estimates. As POR is the preferred measure of association in cross-sectional studies where the disease under study is chronic or has long-lasting risk factors [48]. It was also a better choice over using odds ratios generated using logistic regression, as they tend to overestimate the measure of association when used in cross-sectional studies [49,50].

### Limitations

Data from private schools was collected during exam season, limiting participants’ gaming. Conversely, data from government schools was obtained at start of school year providing ample opportunity for gaming during the preceding summer vacation. This temporal disparity could introduce discrepancy in gaming behavior. Only 18 and 19 year olds were sampled, owing to consent and eligibility constraints. Given that age is a predictor of IGD, exclusion of younger individuals may introduce selection bias. Student clustering within classes could induce data dependency, yielding inaccurate results. However, limited number of clusters and homogenous demographic characteristics of participants from the two schools, could not justify the use of Generalized Estimating Equation to mitigate the effects of clustering. Those who reported playing multiple video games, the highest IARC rating was used for evaluating their exposure status, introducing potential for exposure overestimation.

### Recommendations

Longitudinal studies can help establish causality between IGD and gaming behavior. Schools can raise awareness among students and parents regarding healthy gaming practices of short gaming sessions of less than 3 hours and avoid the introduction of video games from an early age. Government can enforce regulations to monitor and control video games access to age appropriate consumers.

## Conclusion

IGD is an internationally recognized psychiatric condition gradually gaining prevalence among youth. If left unaddressed, it may prevail into adulthood, burdening an already strained healthcare system. Extended gaming sessions and early age of gaming onset are modifiable factors, if managed in adherence to healthy gaming practices can help reduce IGD prevalence among Pakistani adolescents.
